# Burning fuels burns the brain's bioenergetic bridges: On the importance of physiological resilience

**DOI:** 10.1113/EP091424

**Published:** 2023-09-24

**Authors:** Ryan P. Sixtus, Damian M. Bailey

**Affiliations:** ^1^ School of Biomedical Sciences, Sir Martin Evans Building Cardiff University Glamorgan UK; ^2^ Neurovascular Research Laboratory, Faculty of Life Sciences and Education University of South Wales Glamorgan UK

Anthropogenic climate change will be among our greatest challenges this century. At current global temperatures of 1.1°C above pre‐industrial averages, 75% of hot weather extremes and 18% of heavy precipitation events are attributable to global warming (Fischer & Knutti, 2015), and 1‐in‐1000‐year events are as much as 150 times more likely to occur (Philip et al., 2021). With global temperatures predicted to rise from 1.5°C to 4.4°C this century (IPCC, 2023), the frequency and severity of extreme weather events are certain to increase; indeed each successive 5‐year period since the turn of the millennium has recorded a hotter average temperature (World Meteorological Organization, 2013, 2016, 2019). Industrialisation and technological advancements have brought many benefits to health and longevity—from cradle to grave—but these improvements have also swapped out one set of diseases for another. Improved ease of daily life in the developed world has contributed to a burgeoning prevalence of cardio‐cerebro‐metabolic diseases that collectively reduce ‘physiological resilience’ in the face of climatic extremes; that is, pre‐existing disease reduces the capacity of the body to cope with, and respond to, environmental stressors such as excess heat. Here, we briefly consider the concept of physiological resilience in vulnerable populations at the intersection of these current, unprecedented environmental and social challenges.

The combination of rising global temperatures, increased frequency of extreme weather events and lower physiological resilience in vulnerable populations is a recipe for disaster. Elderly populations remain most at risk. A staggering ∼80% of those who died during the 2003 heat wave were aged over 75 years (Fouillet et al., 2008), given the extent of their pre‐existing disease burden (Meade et al., 2020). The presence of cardio‐cerebro‐metabolic disease (a cluster of the most common diseases in developed countries) reduces functional capacity and physiological resilience in the face of extreme temperatures (Kenny et al., 2010). While the elderly are the most commonly studied at‐risk population, we are increasingly aware of populations for whom cardio‐cerebro‐metabolic dysfunction and disease are early life occurrences. These include those born to assisted reproductive technologies or low birth weight (e.g., preterm or growth‐restricted; Crump, 2020; Rimoldi et al., 2015; Sixtus et al., 2023). The Intergovernmental Panel on Climate Change (IPCC) predicts that the main risk for morbidity across the first half of this century will be through climate‐induced exacerbation of health conditions in these vulnerable populations (Smith et al., 2014). Epidemiological evidence from recent heat waves across China, India, America and Australia highlight a wide range of ‘excess’ deaths (deaths above the reference period trend) from 2.5% to 5.0% to as high as 62%, (Anderson & Bell, 2011; Ma et al., 2015; Nitschke et al., 2011; Singh et al., 2020). While heat waves undoubtedly grab sensational headlines—70,000 excess deaths during the 2003 European heat wave (Robine et al., 2008)—the insidious rise in global temperatures may prove equally, if not more costly over time, particularly if worse‐case predictions of 4.4°C are realised (IPCC, 2023). As such, it is imperative that we improve our understanding of vulnerable populations, across the human ageing continuum, and consider targeted countermeasures to optimise risk mitigation.

The brain is a good place to start since it orchestrates the physiological and behavioural responses to multiple environmental stressors, including heat. It is the most sensitive organ to changes in temperature and consequently exerts the most powerful thermoeffector responses (Wang et al., 2014). Much of the thermoeffector response is reliant upon the cardiovascular system, which balances the need for convective and evaporative heat loss while sustaining blood flow and substrate delivery to the brain and essential organs. Regions not essential for the thermoregulatory response, including the brain, are vasoconstricted (Wilson et al., 2006), with resultant oxygen (O_2_) extraction increasing proportionally (Bain et al., 2014). The evolutionary strategy of the brain has been to prioritise substrate (O_2_/glucose) supply over reserve (Bailey, 2019b). As such it is critically dependent on its vascular lifeline and, given its disproportionately elevated bioenergetic demands relative to tissue mass (20–25% of basal metabolic rate for just 2% of total body weight; Leonard et al., 2003), exquisitely vulnerable to failure (Bailey, 2019b).

Elevated oxidative–inflammatory–nitrosative stress (OXINOS) coincides with heat stress, known to impair physiological resilience when in excess subsequent to free radical‐mediated structural destabilisation of cell membranes, scavenging of nitric oxide and corresponding reduction in vascular endothelial function (Bailey, 2019b). As global warming contributes to hotter summers (3 July 2023 recorded the hottest global temperatures yet; Dickie, 2023), OXINOS may predispose to cardio‐cerebro‐metabolic dysfunction in vulnerable populations and tip the scales toward maladaptation. This ‘tipping point’ from the hormetic adaptive towards the OXINOS‐mediated maladaptive phenotype has been observed in lowlanders travelling to high‐altitude suffering from acute mountain sickness (Bailey et al., 2009) and high‐altitude pulmonary oedema (Bailey et al., 2010), to native highlanders suffering from the debilitating syndrome of chronic mountain sickness (Bailey et al., 2019; Stacey et al., 2023). Heat stress and chronically elevated OXINOS ‘consumes’ the physiological reserve, further enhancing cerebral vulnerability (Low et al., 2009; Wilson et al., 2006). Furthermore, it reduces functional reactivity to other environmental stressors.

Emerging evidence indicates that elevated systemic OXINOS is common among many vulnerable populations (e.g., prematurity, cardiovascular disease, ageing). Recent findings in those born preterm or growth‐restricted, as well as to assisted reproductive technologies (accounting for 10% and 2–4% of live births, respectively), indicate that these populations have impaired lifelong cardiovascular function and are potentially at greater physiological risk in the face of climate change (Rimoldi et al., 2015; Sixtus et al., 2023). While not yet linked explicitly to OXINOS, it may contribute, at least in part, to the underlying cardio‐cerebro‐metabolic disease pathophysiology (Bavineni et al., 2019; Dammann & Leviton, 2014; Dinh et al., 2014; Humberg et al., 2020; Sutherland et al., 2014).

Examining the integrated physiological response to stressors beyond our traditional healthy and vulnerable populations may further our understanding of the boundary continuum between adaptation and maladaptation and disassociate physiologically hormetic from pathologically damaging OXINOS. In this, our fellow species adapted to the extremes (Nature's so‐called ‘platinum performers’; see Bailey, 2019a), unified by their ability to suppress OXINOS in environmental extremes, may provide precious clues as to how we might ‘better’ adapt to our rapidly changing world. With time, we too as humans will have to become extreme performers, albeit constrained by a less developed evolutionary ‘toolkit’. Examples of exceptional adaptations to which we can only marvel include the crucian carp (*Carassius Carassius*), which can withstand up to ∼5 months of anoxia (Nilsson, 2001), or the camel (*Camelus dromedarius*), which can tolerate prolonged exposure to temperatures of ∼55°C and up to 14 days’ water deprivation (Elkhawad, 1992). By understanding the specialist adaptations exploited by Nature's ‘extremophiles’, be it impressive energy and water stores or specialised membrane and redox homeostasis, we may discover novel therapeutic targets to potentially improve physiological resilience in the lesser mortal vulnerable populations.

Our own evolutionary past was shaped by climatic pressures. Chief among hominin adaptations was the expansion of the brain (3.5‐fold beginning ∼3–2 Mya) and associated technological advancements (e.g., the Acheulean axe, ∼1.7 Mya). This adaptation for making and refining technologies has continued our expansion to new terrestrial environments and even into space. As our brain committed to the strategy of substrate supply over reserve, the vascular network supplying the brain increased 6‐fold with multiple redundancies to preserve this supply (Seymour et al., 2016). However, this assumes that perfusion is inexhaustible, which is not the case among vulnerable heat‐stressed populations. Perfusion commonly fails during heat waves (usually with a heart attack or stroke; Campbell et al., 2018; Moraes et al., 2022), but with global warming‐induced seasonal extremes we speculate that systemic or localised OXINOS will play an increasingly important role as vulnerable populations exceed their physiological reserves (see also Pallubinsky (2021) on thermal resilience outside the thermal comfort zone, and Tipton and Montgomery (2022) on climate change and healthy ageing). So, the question remains, can we learn in the face of this new challenge? Can we learn from our own species—both our evolutionary ancestors and those facing future challenges—or are there answers to be gleaned from other species? These questions are necessary, not just at the intersection of climate change challenges and vulnerable populations, but also as we look to adapt to new challenges such as deep space exploration.

Countries around the world (e.g., Portugal, England, Austria, Macedonia) continue to instigate public health action plans for climate change—and heat waves in particular—but we are missing one key ingredient. For many of our vulnerable populations, including those with cardio‐cerebro‐metabolic disease, there are gaping holes in our knowledge of their integrated, systemic response to heat stress. This makes it difficult to compile adequate action plans for populations outside the elderly. Further still, we lack the capacity to inform these populations of when or how they are at risk during high temperatures—or even *who* may be at risk! We have highlighted two potentially vulnerable populations seemingly outside our typical understanding of who is at risk during high‐temperature days, but there are many populations (e.g., those with obesity, diabetes mellitus, metabolic syndrome) who have altered functional capacity in the context of climate change. The quandary here, is that without physiological quantification to steer clinical decision‐making, many vulnerable populations remain unrecognised in the climate change context and uninformed of their vulnerability. That carbon dioxide levels are inexorably rising – and oxygen predicted to gradually wither way over time (See editorial by Bailey & Poole, 2022) – only as more fuel to these burning physiological fires. Interrogating physiological resiliency at the intersection of vulnerability and climate change will better inform our public health action plans—at all levels—to protect our populations in this changing world.

## AUTHOR CONTRIBUTIONS

R.P.S. and D.M.B. are jointly responsible for conception, drafting and critically revising the manuscript. Both authors approve of the final version of the manuscript and agree to be accountable for all aspects of the work, ensuring that questions related to the accuracy or integrity are appropriately investigated and resolved. All persons designated as authors qualify for authorship, and all those that quality are listed.

## CONFLICT OF INTEREST

The authors declare no conflicts of interest.

## FUNDING INFORMATION

R.P.S. is supported by a BBSRC project grant (BB/V014765/1). D.M.B. is supported by a Royal Society Wolfson Fellowship (WM1700007).
